# Advances in Photocatalytic CO_2_ Reduction with Water: A Review

**DOI:** 10.3390/ma10060629

**Published:** 2017-06-08

**Authors:** Samsun Nahar, M. F. M. Zain, Abdul Amir H. Kadhum, Hassimi Abu Hasan, Md. Riad Hasan

**Affiliations:** 1 Sustainable Construction Materials and Building Systems (SUCOMBS) Research Group, Universiti Kebangsaan Malaysia (UKM), UKM Bangi 43600, Malaysia; riad.hasan@siswa.ukm.edu.my; 2 Department of Architecture, Universiti Kebangsaan Malaysia (UKM), UKM Bangi 43600, Malaysia; 3 Department of Chemical & Process Engineering, Universiti Kebangsaan Malaysia (UKM), UKM Bangi 43600, Malaysia; amir8@ukm.edu.my (A.A.H.K.); hassimi@ukm.edu.my (H.A.H.); 4 Department of Civil & Structural Engineering, Universiti Kebangsaan Malaysia (UKM), UKM Bangi 43600, Malaysia

**Keywords:** photocatalysis, CO_2_ reduction, visible light irradiation, plasmonic photocatalyst, surface plasmon resonance (SPR)

## Abstract

In recent years, the increasing level of CO_2_ in the atmosphere has not only contributed to global warming but has also triggered considerable interest in photocatalytic reduction of CO_2_. The reduction of CO_2_ with H_2_O using sunlight is an innovative way to solve the current growing environmental challenges. This paper reviews the basic principles of photocatalysis and photocatalytic CO_2_ reduction, discusses the measures of the photocatalytic efficiency and summarizes current advances in the exploration of this technology using different types of semiconductor photocatalysts, such as TiO_2_ and modified TiO_2_, layered-perovskite Ag/ALa_4_Ti_4_O_15_ (A = Ca, Ba, Sr), ferroelectric LiNbO_3_, and plasmonic photocatalysts. Visible light harvesting, novel plasmonic photocatalysts offer potential solutions for some of the main drawbacks in this reduction process. Effective plasmonic photocatalysts that have shown reduction activities towards CO_2_ with H_2_O are highlighted here. Although this technology is still at an embryonic stage, further studies with standard theoretical and comprehensive format are suggested to develop photocatalysts with high production rates and selectivity. Based on the collected results, the immense prospects and opportunities that exist in this technique are also reviewed here.

## 1. Introduction

Global warming is viewed to be one of the vital environmental concerns that humankind is dealing with [1]. CO_2_ contributes mostly to the worldwide climate change because it is more than 64% effective than other greenhouse gasses in the atmosphere [2]. This chemically stable gas contributes to the increase in global temperature through absorption and re-emission of infrared radiation. In the past century, the temperature of the Earth’s surface increased by roughly 0.6 K; the warming trend reveals more significant changes in last 20 years, according to the Intergovernmental Panel on Climate Change (IPCC) [3]. The consequences of the greenhouse effect are global and severe, such as ice melting at the Earth’s poles, the quick rising of sea level, and growing precipitation across the globe [4]. To deal with these issues, numerous studies have been conducted over the last few decades applying various strategies to control CO_2_ emission or convert it into other products.

There are at least three routes of lowering the amount of CO_2_ in the atmosphere: (i) direct reduction of CO_2_ emission; (ii) CO_2_ capture and storage (CCS); and (iii) CO_2_ utilization [5,6,7]. Lowering the CO_2_ emission may seem quite unrealistic because of the present human lifestyle and emergent use of fossil fuel. The potential of CCS technology can be restrained because of the environmental risk of leakage and the energy requirement for fuel compression and transportation. Among the renewable resources, solar energy is the most exploitable one by making available more energy to the Earth for every hour than the total amount of energy humans consume in a year [8].

Harvesting this abundant sunlight in solving environmental problems is a promising approach and one of the ultimate goals for sustainability of global development. In the long term, photocatalytic conversion of CO_2_ utilizing solar energy is the most appealing route for CO_2_ reduction [9,10]. In addition, CO_2_ reduction advances recycling of carbon source [8]. The interest in this field of research has begun with the work of Fujishima and Honda in 1972 [11]. The advancements in nanotechnology, particularly the synthesis of nanomaterials with different structures and morphologies [12,13], and the most recent approach of using noble metals, such as Au or Ag, with surface plasmon resonance (SPR) to enhance the photocatalytic efficiency of TiO_2_ or other semiconductors [14,15,16] have facilitated the progress.

For real life application, a photocatalytic system must be capable of working under daylight even when the sun is not directly overhead and show both long-time consistency and efficiency. The reduction process has to be promoted while suppressing any side reaction that can occur during the reaction, and H_2_O should be used as an electron source [17]. Unfortunately, a photocatalyst that satisfies all these requirements has not been reported yet. A considerable number of review papers on this emerging topic have already been published. Some papers focus on the advances in developing novel photocatalysts with high photocatalytic activity [18,19,20,21,22], while others on studying the enhancement mechanisms and the influences of co-catalysts [23], the applications by highlighting on the reaction conditions, reactor design and analysis methods [18,24] and comprehensive discussion on general considerations that apply specifically to CO_2_ reduction [25,26]. Furthermore, extensive studies on TiO_2_-based photocatalysts [27,28,29] and noble metal nanoparticles dispersed plasmonic photocatalysts have been published as well [30,31,32]. Nevertheless, the basic insight of photocatalytic CO_2_ reduction in presence of H_2_O and comparison among the photocatalytic efficiency of different photocatalysts in this reaction has not been clearly documented to date. This review paper covers the basic aspects of photocatalytic CO_2_ reduction process with H_2_O, concentrating on recently reported semiconductor photocatalysts with high photoactivity, particularly on plasmonic photocatalysts.

## 2. Photocatalysis and Photocatalytic Reduction of CO_2_ with H_2_O

The word photocatalysis consists of two parts: photo and catalysis, “photo” means light and “catalysis” is the performance of a substance during the chemical transformation of the reactants to modify the reaction rate without being changed ultimately [33]. In practice, the word photocatalysis refers to the acceleration of a photoreaction in the presence of a catalyst [34]. In photocatalytic CO_2_ reduction system with water, both photo-reduction of CO_2_ and photo-oxidation of H_2_O occur simultaneously under sunlight irradiation using a suitable photocatalyst. A variety of reaction conditions intensely affects the product distribution of this reaction, such as reactor geometry, catalyst type, sacrificial reagents, and even illumination type. Thus, predicting the product distribution of a particular photocatalytic reaction is very challenging [35].

The photocatalytic CO_2_ reduction is a very effective method considering that no additional energy is needed and no negative effect on the environment is produced. The use of cheap and abundant sunlight to transform this major greenhouse gas into other carbon containing products is also an ideal approach because of its low cost. Here, the high activation energy to break very stable CO_2_ molecule is provided by solar energy [35]. To date, many photocatalysts, including oxides and non-oxides, e.g., TiO_2_, ZnO, Fe_2_O_3_, ZrO_2_, SnO_2_, BiWO_3_, Ti-MCM-41, CdS, TNTs, ZnS, GaN, and SiC, have been studied for the photocatalytic reduction of CO_2_ with H_2_O. A summary of different photocatalytic systems employed in this technology since 2010 are given in Table 1.

One of the major obstacles to this research progress is that most of the CO_2_ reducing photocatalysts are not visible light responsive [63]. In this context, numerous types of photocatalysts have been developed. A few of these catalysts performed under visible light irradiation with high conversion rate and selectivity, whereas other catalysts were weakly responsive under visible light and showed a low rate of reaction yield [64]. The introduction of plasmonic metal onto semiconductor materials to enhance photocatalytic activity has been demonstrated to be very attractive in the visible region.

In the following sections, the basic mechanisms and principles of measuring the efficiency of a photocatalyst in photocatalytic CO_2_ reduction with H_2_O are discussed.

### 2.1. Theoretical Approach

Photocatalysis means activating a semiconductor using sunlight or artificial light. When a semiconductor material absorbs photons of sufficient energy, its electrons are excited from the valence band (VB) to the conduction band (CB), creating electron–hole pairs. VB is the highest energy band occupied by electrons and CB is the lowest energy band in which there is no electron at the ground state [65]. These photo-generated electrons can move to the surface of a semiconductor and react with the adsorbed species on the surface. Meanwhile, electron–hole recombination is also possible [66]. The efficiency of the photocatalytic reaction depends on the competition between these two processes [67].

The basic photocatalytic process can be summarized as follows:

(i) Absorption of photons with suitable energy and generation of electron–hole pairs;
(ii) Separation and transportation of electron–hole pairs (charge carriers); and
(iii) The chemical reaction of surface species with charge carriers [68,69].

**Figure 1:**
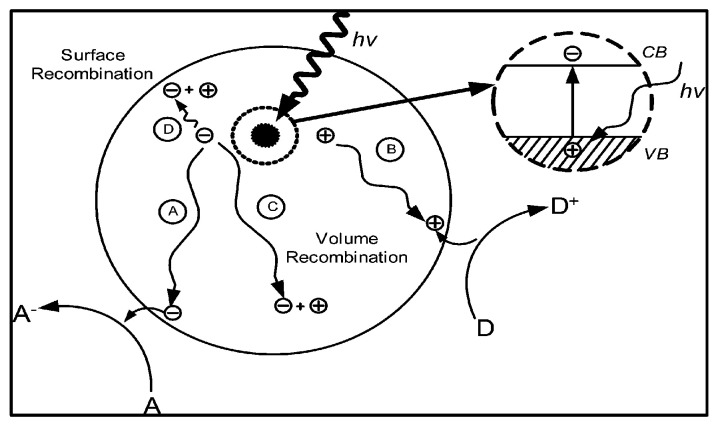
Schematic diagram of photo-excitation and electron transfer process (adapted from [63]).

This process is illustrated in Figure 1. As the charge recombination process (~10^−9^ s) is usually much faster than the reaction process (~10^−3^–10^−8^ s), acceleration of the electron–hole separation step remarkably affects the reaction yield [22].

Apart from the direct photon-excited charge carrier generation process in semiconductors Figure 1, collisions, photon-electron interaction [70,71,72] or electron transfer from the SPR-excited metal nanoparticle [73,74] can also generate electron–hole pairs. However, all of the photo-excited electrons reaching the surface cannot reduce thermodynamically inert and very stable CO_2_ compound. This reduction reaction is endergonic and requires both hydrogen and energy [19]. Thus, photocatalytic CO_2_ reduction using sunlight and water has the potential to be the most feasible means to remove atmospheric CO_2_.

The reduction potential for the various products of CO_2_ reduction at pH 7 is presented in Table 2. On the one hand, single-electron CO_2_ reduction reaction requires a highly negative potential of −1.9 eV, which makes the one-electron reduction process very unfavorable. On the other hand, the proton assisted multi-electron CO_2_ reduction reaction requires comparatively low redox potential (Table 2) and are more favorable. Photocatalysts can facilitate these reduction processes with lower potential. For this purpose, an ideal photocatalyst generally requires two characteristics: (i) the redox potential of the photo-excited VB hole must be sufficiently positive so that the hole can act as an electron acceptor; and (ii) the redox potentials of the photo-excited CB electron must be more negative than that of the CO_2_/reduced-product redox couple.

**Figure 2:**
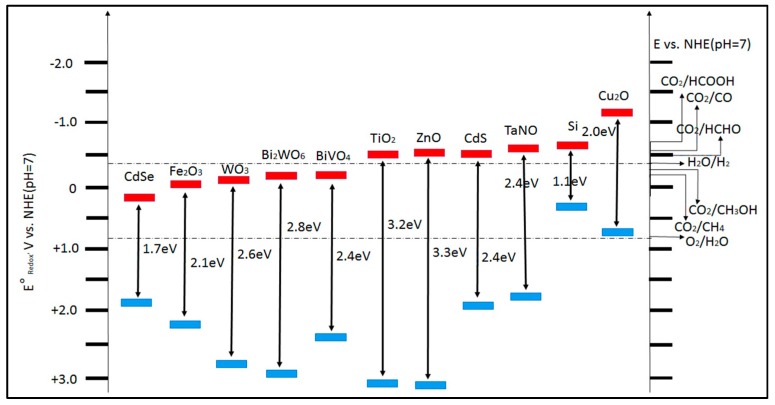
Schematic representation of conduction band, valence band potentials, and band gap energies of various semiconductor photocatalysts and relative redox potentials of the compounds involved in CO_2_ reduction at pH 7 (Adapted from [22]).

Upon absorbing radiation from the light source, photo-generated holes in the VB of the photocatalyst oxidize H_2_O. In addition, the photo-generated electrons in its CB form products such as HCOOH, HCHO, CH_3_OH, and CH_4_, by reducing CO_2_. Here, the relation between the energy levels of the photocatalyst and the redox agent determines the type of reaction that takes place. Figure 2 shows the CB, VB potentials, and bandgap energies of various semiconductor photocatalysts and relative redox potentials of compounds involved in CO_2_ reduction. The final carbon containing products are determined by the specific mechanism to conduct the reaction. The number and rate of transferred electrons from the photo-generated carriers to the reaction species in the reaction system also contribute in this process [26].

The most commonly used light source for photocatalysis is ultraviolet (UV) light. The high energy content of UV light can effectively excite most photocatalysts. Thus, the majority of publications on photocatalytic CO_2_ reduction processes are still based on using artificial UV light from high-power lamp [75,76,77]. Only about 4% of solar energy is used by UV light where 43% of solar energy is occupied by visible light; thus, a photocatalyst with a narrow bandgap that can use visible light is in high demand [65,78]. At present, a significant number of studies focus on the direct use of visible light both from artificial and natural sources. Using visible light is more favorable than using UV light because visible light is readily available from sunlight. However, the energy content of visible light is less competitive compared to UV light. Thus, in photocatalytic reduction, the visible light might not provide for an adequate amount of energy for photo-excitation of the catalysts. As such, photocatalysis using visible light and sunlight faces a great challenge [79].

### 2.2. Measures of Photocatalytic Efficiency

The photocatalytic CO_2_ reduction efficiency is generally measured by the yield of the product. Here, the general unit for *R* is mol·h^−1^·g^−1^ of catalyst and for the product either in molar units (μmol) or in concentration units (ppm).

(1) $$R=\frac{n\left(\mathrm{Product}\right)}{\mathrm{Time}\times m\left(\mathrm{Catalysts}\right)}$$

In the catalyst-based measurements, the efficiency of the photocatalyst usually depends on the amount of photocatalyst, the intensity of the light, lighting area, etc., so under the irradiation of light, the amount of product formed by per gram of photocatalyst within a certain time period can be measured by its apparent quantum yield. It is calculated by using the amount of product and the incident photon number as shown in the following equations [19,26]. When the photocatalytic reduction reaction gives complex products, then the number of reacted electrons in the equation denotes the sum of the reacted electron to form each product [80,81]. Thus, in light-based measurements, the quantum yield of CO_2_ photo-reduction into different products can be calculated using following equations:

(2) $$\mathrm{Overall}\;\mathrm{quantum}\;\mathrm{yield}\left(\%\right)=\frac{\mathrm{Number}\;\mathrm{of}\;\mathrm{reacted}\;\mathrm{electrons}}{\mathrm{Number}\;\mathrm{of}\;\mathrm{absorbed}\;\mathrm{photons}}\times \;100\%$$

(3) $$\mathrm{Apparent}\;\mathrm{quantum}\;\mathrm{yield}\left(\mathrm{QY},\%\right)=\frac{\mathrm{Number}\;\mathrm{of}\;\mathrm{reacted}\;\mathrm{electrons}}{\mathrm{Number}\;\mathrm{of}\;\mathrm{incident}\;\mathrm{photons}}\times \;100\%$$

(4) $$\left(\mathrm{Apparent}\right)\;\mathrm{quantum}\;\mathrm{yield}\;\mathrm{of}\;\mathrm{CO}(\%)\;=\;\frac{2\times \mathrm{Number}\;\mathrm{of}\;\mathrm{CO}\;\mathrm{molecules}}{\mathrm{Number}\;\mathrm{of}\;\mathrm{incident}\;\mathrm{photons}}\times \;100\%$$

(5) $$\left(\mathrm{Apparent}\right)\;\mathrm{quantum}\;\mathrm{yield}\;\mathrm{of}\;\mathrm{HCOOH}\left(\%\right)=\frac{2\times \mathrm{Number}\;\mathrm{of}\;\mathrm{HCOOH}\;\mathrm{molecules}}{\mathrm{Number}\;\mathrm{of}\;\mathrm{incident}\;\mathrm{photons}}\times \;100\%$$

(6) $$(\mathrm{Apparent})\;\mathrm{quantum}\;\mathrm{yield}\;\mathrm{of}\;\mathrm{HCHO}(\%)\;=\frac{4\times \mathrm{Number}\;\mathrm{of}\;\mathrm{HCHO}\;\mathrm{molecules}}{\mathrm{Number}\;\mathrm{of}\;\mathrm{incident}\;\mathrm{photons}}\times \;100\%$$

(7) $$(\mathrm{Apparent})\;\mathrm{quantum}\;\mathrm{yield}\;\mathrm{of}\;CH_{3}\;\mathrm{OH}(\%)\;=\frac{6\times \mathrm{Number}\;\mathrm{of}\;CH_{3}\;\mathrm{OH}\;\mathrm{molecules}}{\mathrm{Number}\;\mathrm{of}\;\mathrm{incident}\;\mathrm{photons}}\times \;100\%$$

(8) $${(\mathrm{Apparent})\;\mathrm{quantum}\;\mathrm{yield}\;\mathrm{of}\;\mathrm{CH}}_{4}(\%)\;=\frac{{8\times \mathrm{Number}\;\mathrm{of}\;\mathrm{CH}}_{4}\;\mathrm{molecules}}{\mathrm{Number}\;\mathrm{of}\;\mathrm{incident}\;\mathrm{photons}}\times \;100\%$$

## 3. Recent Photocatalysts for CO_2_ Reduction with H_2_O

The first step towards enhancing the photocatalytic activity is the selection of a proper photocatalyst. It is a subject of considerable importance both for practical application of photocatalysts and understanding their mechanism. Photocatalysts could be categorized into two basic groups based on their structures: homogeneous and heterogeneous photocatalysts.

The seminal work by Lehn et al. demonstrated the selective CO_2_ reduction into CO by using Re(I) diimine complexes [82]; since then, the use of metal complexes in photocatalysis has been greatly studied for both CO_2_ reduction [83,84,85,86] and H_2_O oxidation [87,88,89]. CO_2_ is efficiently reduced to form CO when homogeneous photocatalysts, such as Re complexes, are used in the presence of electron donors, such as triethanolamine [80,90,91]. However, CO_2_ reduction and H_2_O oxidation processes require distinct reaction conditions.

**Figure 3:**
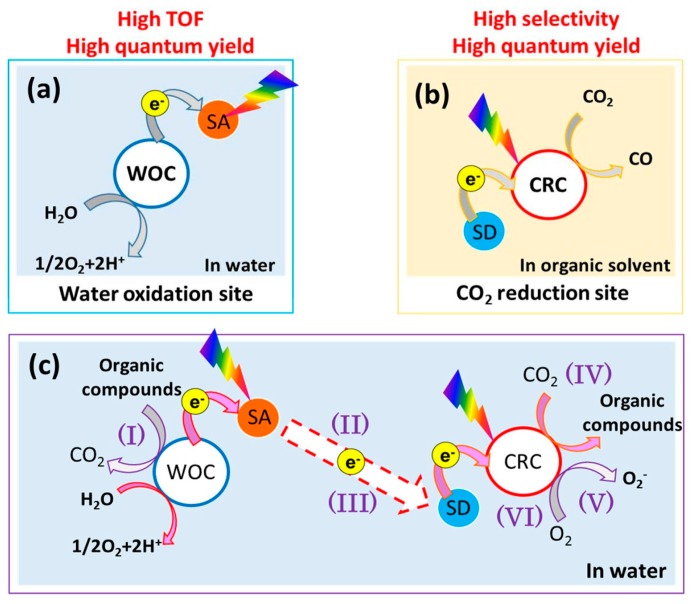
Advantages and disadvantages of metal complex catalysts for CO_2_ reduction with H_2_O oxidation (adapted from [8]). (**a**) The advantages of H_2_O oxidation of a metal complex catalyst (H2O oxidation site) with a sacrificial electron acceptor (SA); (**b**) the advantages of CO_2_ reduction for a metal complex catalyst (CO_2_ reduction site) with a sacrificial electron donor (SD); (**c**) the problems encountered when combining H_2_O oxidation site and CO_2_ reduction site.

As a result, carrying out both of the reaction simultaneously using a metal complex catalyst in a single system is a very difficult task. Reverse oxidation of organic products generated from the reduction of CO_2_ and the reverse reduction of O_2_ generated from the oxidation of H_2_O terminate the continuity of the reaction. Figure 3 summarizes these cases briefly [8]. Figure 3a shows the advantages of H_2_O oxidation of a metal complex catalyst (H_2_O oxidation site) with a sacrificial electron acceptor (SA). Figure 3b shows the advantages of CO_2_ reduction for a metal complex catalyst (CO_2_ reduction site) with a sacrificial electron donor (SD). Figure 3c shows the problems encountered when combining H_2_O oxidation site and CO_2_ reduction site: (I) reverse oxidation of products such as organic compounds; (II) electron transfer from H_2_O oxidation site to CO_2_ reduction site; (III) need to be electron storage; (IV) need to be active in H_2_O; (V) easier reduction of O_2_ than CO_2_; and (VI) stability in H_2_O [8]. A number of challenges are encountered in constructing a homogeneous metal complex system for CO_2_ reduction along with H_2_O oxidation. The inefficient electron transport between reduction and oxidation catalysts is one of the major difficulties in this process. Another drawback is the short lifetimes of the one-electron-reduced species and the photo-excited state in the presence of O_2_ generated by H_2_O oxidation.

Since the pioneering work of Fujishima, Honda, and their co-workers, where they reported the photocatalytic reduction of CO_2_ to organic compounds, such as HCOOH, CH_3_OH, and HCHO, in the presence of various semiconductor photocatalysts, such as TiO_2_, ZnO, CdS, SiC, and WO_3_ [92], many heterogeneous semiconductor compounds, including metal oxides, oxynitrides, sulfides, and phosphides, had been investigated for this purpose [10,20]. TiO_2_, BaLa_4_Ti_4_O_15_, SrTiO_3_, WO_3_ nanosheet, NaNbO_4_, KNbO_4_, Sr_2_Nb_2_O_7_, Zn_2_GeO_4,_ and Zn_2_SnO_4_ are the leading compounds in this list of photocatalysts and the list is increasing enormously in the last five years [1,9,10,18,19,20,28,64,65,93,94,95,96,97,98]. Activation of an inert molecule such as CO_2_ requires contributions of both incident photons and effectively excited electrons. Thus, the presence of reducing agents can assist the CO_2_ activation process. It takes advantage of H_2_O oxidation and CO_2_ fixation when H_2_O is used as the reducing agent. Appropriate incident light and suitable semiconductor materials have an important role in attaining this process. Moreover, intensified processing and sensibly engineered strong catalyst with great accessibility are essential to activate the very small molecules under ambient conditions [99]. Some of the desirable properties of an efficient heterogeneous photocatalyst are a high surface area, single site structure, light absorption, an efficient and long lifetime of charge separation, the high mobility of charge carriers, and product selectivity [25].

Table 1 shows the studies on photocatalytic CO_2_ reduction with H_2_O to obtain good efficiency and selectivity for specific products. However, this approach is still far from practical implementation. Application of photocatalysis in the environmental and energy industries on a large scale is still limited. Among several difficulties in the heterogeneous photocatalysis, the two major ones are low photocatalytic efficiency and the lack of suitable visible-light-responsive photocatalyst [100,101]. The first one is mostly because of the recombination of photo-generated electrons and holes. For example, the most widely used semiconductor photocatalyst, i.e., TiO_2_, is well known for its low cost, nontoxicity, and stability with outstanding optical and electronic properties [102,103], but the high recombination rate of photoexcited electron–hole pairs in TiO_2_ hinders its advanced application [104,105]. Another difficulty is that most of the commonly used photocatalysts like TiO_2_ and ZnO have large band-gaps, so they can only absorb sunlight in the near UV region. Thus, only a small percent of the solar spectrum is utilized, where many low-bandgap photocatalysts, such as CdS and Fe_2_O_3_, show low stability [30]. To resolve these drawbacks, new and more efficient visible-light-active photocatalysts have been studied to satisfy the necessity of future environmental and energy technologies driven by solar power [106]. The development of latest technological advances [82], application of modern synthesis methods to form high-surface-area catalyst nanostructures [83], studies on new co-catalysts to coupled with existing photocatalysts, and investigation on the visible-light-responsive plasmonic photocatalysts are some of the progressing ways of enhancing the photocatalytic activity.

In this section, we present a brief and necessary description on several recently reported semiconductor photocatalysts that exhibit high catalytic activity towards CO_2_ reduction with H_2_O. We limit our discussion here to extensively studied TiO_2_ and modified TiO_2_ photocatalysts, layered-perovskite photocatalyst ALa_4_Ti_4_O_5_, and ferroelectric photocatalyst LiNbO_3_ and presented an overview of visible-light-active novel plasmonic photocatalysts in the next section.

### 3.1. TiO_2_ and Modified TiO_2_

TiO_2_ and modified TiO_2_ composites are the most commonly used photocatalysts worldwide. In TiO_2_-based materials CO_2_ reduction with H_2_O involves these basic six steps: (i) adsorption of the reactants on the photocatalyst; (ii) activation of the adsorbed reactants by photo-generated charge carriers; (iii) surface intermediates formation; (iv) intermediates to products conversion; (v) desorption of the products from the catalyst surface; and (vi) catalyst regeneration. The dynamics of the reaction process and final products from CO_2_ reduction are determined by each of these steps. Previous literature has demonstrated that activation and dissociation process of CO_2_ on TiO_2_ surface can be increased by creating defect on the catalyst surface (e.g., Ti^3+^ and oxygen vacancy). By tailoring the crystal phase of TiO_2_ (e.g., a mixture of anatase/brookite or anatase/rutile), engineering the defects in TiO_2_ and incorporating modifiers with TiO_2_ (e.g., metals, metal oxides, graphene, quantum dot sensitizers); the rate of charge separation and transfer can be enhanced [29].

Most studies in this field adopted a solid–liquid interface reaction mode. In such case, particles of a photocatalyst are dispersed or suspended in the aqueous solution, which dissolves CO_2_. A limited reduction of CO_2_ and preferential adsorption of H_2_O on catalyst surface could occur due to limited solubility of CO_2_ in H_2_O and direct contact of liquid H_2_O with the photocatalyst [22]. These limitations could be overcome by using solid–gas or solid–vapor mode of reaction, which can also increase the reduction of CO_2_. For example, Xie et al. showed that the rate of hydrocarbon product formation increases by more than three times along with decreasing H_2_ production from H_2_O when TiO_2_ (P25) or Pt-TiO_2_ photocatalyst is placed on a holder surrounded by gaseous CO_2_ and H_2_O instead of dispersing the photocatalyst in liquid water (Table 3) [107]. The CO_2_ reduction selectivity increased pronouncedly from 11–19% to 40–56%. Thus, the solid–vapor reaction mode is better for preferential reduction of CO_2_ in the presence of H_2_O. The microstructure of the photocatalysts and the ratio of gaseous CO_2_ and H_2_O in gas mode reaction influence the photoactivity and selectivity. For example, Zhang and co-workers found an increased CH_4_ formation on Pt-loaded TiO_2_ nanotubes with increasing concentration of H_2_O molecules surrounding the TiO_2_ nanotubes, as well as a high concentration of –OH groups on the surface. However, the ratio of gaseous CO_2_/H_2_O displayed little effect on product formation over Pt-TiO_2_ nanoparticles [108]. These results indicated that the adsorption of H_2_O molecules on the photocatalyst surface can affect the photoreduction in gas mode. Optimizing and modulating the microstructure and surface property of the semiconductor is a very effective way to improve the activity and selectivity of photocatalytic CO_2_ reduction in gas mode [19].

The light source also has a strong impact on this reduction process. Varghese et al. reported that the rate of product formation from CO_2_ reduction is at least 20 times higher under outdoor sunlight than previously published reports, where photocatalytic reductions were carried out using UV illumination [109]. Even though TiO_2_ is the most widely studied and used photocatalyst, in spite of its high conversion rate, the overall quantum yield is considerably low for the reactions that have been studied. Certainly as low as 10% for most processes [110]. Pure TiO_2_ shows a lower efficiency towards the reduction reaction due to its high rate of charge-carriers recombination and a shorter lifetime of photo-generated charges. So far, many efforts are been made to utilize this photocatalyst more efficiently including nanostructured TiO_2_ synthesis, single crystal TiO_2_, metal or non-metal doped TiO_2_, dye-sensitized TiO_2_ etc. The majority of these techniques are expensive at the same time very complex.

### 3.2. Ag co-Catalyst Loaded ALa_4_Ti_4_O_5_ (A = Ca, Sr, and Ba)

In recent years, a new set of materials unrelated to TiO_2_ emerged in the photocatalysis study. Layered-perovskite ALa_4_Ti_5_O_15_ (A = Ca, Sr, and Ba) photocatalysts with 3.79–3.85 eV of bandgaps, had been previously reported for effective water splitting [111] and later on also employed for the CO_2_ reduction by Iizuka et al. In this process, HO^−^ was used as a reducing reagent. They also discussed the factors affecting the photoactivity on the basis of the examination and characterization of the co-catalysts [39]. They found that Ag co-catalyst-loaded ALa_4_Ti_4_O_15_ (A = Ca, Sr, and Ba) reduces CO_2_ into CO using H_2_O as an electron donor. For this purpose, Ag co-catalyst-loaded BaLa_4_Ti_4_O_15_ was the most effective photocatalyst.

Although large amounts of reacted electrons and holes are present (in the order of Au > Cu > Ru > NiO_x_ > Ag), Ag is the most active co-catalyst for CO_2_ reduction, and its photocatalytic activity showed dependence on the loading methods (Table 4 and Table 5). In addition to the size of Ag particles, the unique location of Ag nanoparticles on the working photocatalyst is also important. By a chemical reduction method, Ag particles could be loaded both on the edge and the basal planes of BaLa_4_Ti_4_O_15_, which had a plate morphology with ~100 nm thickness and ~1 μm width. This liquid-phase chemical reduction method is the best for loading fine Ag particles, where the condition of the Ag co-catalysts is changed at the beginning stage of the photocatalytic reaction.

In the photo-deposition process, Ag particles of 30–40 nm size are loaded on the edge of the plate predominantly. The photo-generated holes could dissolve the Ag particles on the basal plane, which are then re-photo-deposited on the edge during the photocatalytic reaction. In this case, the re-photo-deposited Ag particles on the edge are smaller than 10 nm and more uniform than the direct photo-deposited Ag particles [39]. The Ag-loaded BaLa_4_Ti_4_O_15_ prepared by impregnation followed by H_2_ reduction also shows the re-photo-deposition, and the sizes of the re-photo-deposited Ag particles are within 10–20 nm. The CO formation rate in BaLa_4_Ti_4_O_15_ under working conditions shows a change in an opposite sequence of the order of Ag particles size on its edge plane. Thus, smaller Ag particles on the photocatalyst result in higher activity of CO formation. The CO_2_ reduction mainly occurred on the Ag nanoparticle loaded edge of the BaLa_4_Ti_4_O_15_ plate, whereas H_2_O oxidation occurred on the basal plane. The unique location of Ag nanoparticles on the photocatalyst can separate the plane of oxidation and reduction reaction, thus increasing the activity of photocatalytic CO_2_ reduction [107]. This Ag-doped system shows high selectivity for CO_2_ reduction as indicated by the ratio of CO/H_2_ (~2.0), but solar energy conversion rate is very low due to the large bandgap of this catalyst [8].

### 3.3. Ferroelectric LiNbO_3_

The use of substances with a dipole, which separates the photogenerated electrons and holes, is an important part of surface photochemistry that has not been largely addressed. These substances are ferroelectric materials. The selective oxidation and reduction reactions, which take place on the surface of BaTiO_3_, was demonstrated by an early work of Giocondi and Rohrer [112]. Subsequent work on the ferroelectric methods PbZr_0.3_Ti_0.7_O_3_ [113,114] and LiNbO_3_ [115] indicated that the dipole in the ferroelectric material determines the space charge layer structure because of the spontaneous polarization associated with lattice distortions. Ferroelectric LiNbO_3_ is a promising photocatalyst for CO_2_ reduction due to its comparatively strong remnant polarization of 70 μC/cm^2^ [116] than other materials, such as KNbO_3_ (30 μC/cm^2^) [117] and lead zirconate titanate [Pb(Zr_x_Ti_1−x_)O_3_ (PZT)] (25 μC/cm^2^) [118]. In spite of the wide bandgap of LiNbO_3_, which is 3.78 eV, its high remnant polarization was exploited to achieve products from CO_2_ conversion either under high-pressure mercury lamp illumination or natural sunlight [119]. In the case of solid–liquid reactions, LiNbO_3_ shows low efficiency in CO_2_ reduction [120] but in a solid–gas reaction scheme, this ferroelectric material produces seven times the product formed by TiO_2_ under UV light; under visible light, 36 times more product are produced compared with that by TiO_2_. This high rate of product formation by LiNbO_3_ in CO_2_ reduction with H_2_O can be explained by its strong remnant polarization, which is absent in TiO_2_.

Remnant polarization creates an electric field in ferroelectric materials, like LiNbO_3_, which is similar to usual p–n junction electric field. This electric field separates the photo-excited electrons and holes, leading to an enhanced lifetime of carriers. Thus, photo-excited carriers participate more in redox reactions because there is less chance of charge recombination [121]. In LiNbO_3_, the decay time of polaron photo-luminescence is very high (9 μs) [122], thus confirming the controlled charge recombination and longer lifetime of carriers. Remnant polarization also causes a charge experienced at the interface of LiNbO_3_, which interacts with the species in contact with the surface, thereby creating a strongly bound layer [123] and altering the bonding nature in physisorbed materials. Matt et al. suggested that in previous liquid–solid reaction schemes, particularly this tightly bound layer hinders high level of product formation. Another reason of high product formation rate by LiNbO_3_ could be the more energetically favorable reaction pathway availability than those from lower-energy photo-excited electrons of semiconductors [121]. For these characteristics, LiNbO_3_ is considered as a promising photocatalyst in concrete construction to improve air quality [124]. An experimental demonstration of this effect was carried out by Nath et al., in which the addition of LiNbO_3_ to concrete materials reduces CO_2_ in the presence of water and forms O_2_ [125]. This relatively new compound has already been used in electronic instruments in place of TiO_2_ to achieve artificial photosynthesis [126]. These studies clearly showed that ferroelectric LiNbO_3_ is effective even under weak solar energy in the ambient atmosphere for CO_2_ reduction with H_2_O. Further studies are required for the large-scale use of this water-insoluble, chemically inert photocatalyst.

## 4. Plasmonic Photocatalyst

For highly efficient photocatalysis process, plasmonic photocatalysts have become a topic of increasing interest, in recent years [101,103,127,128,129,130,131]. Nanoparticles of noble metals like Au, Ag, Pt exhibit strong absorption in the UV-visible region due to their surface plasmon resonance (SPR) [132]. SPR simply means the collective oscillations of conduction band electrons in a metal particle and it is driven by the electromagnetic field of incident light [133,134]. It is also known as localized surface plasmon resonance (LSPR).

**Figure 4:**
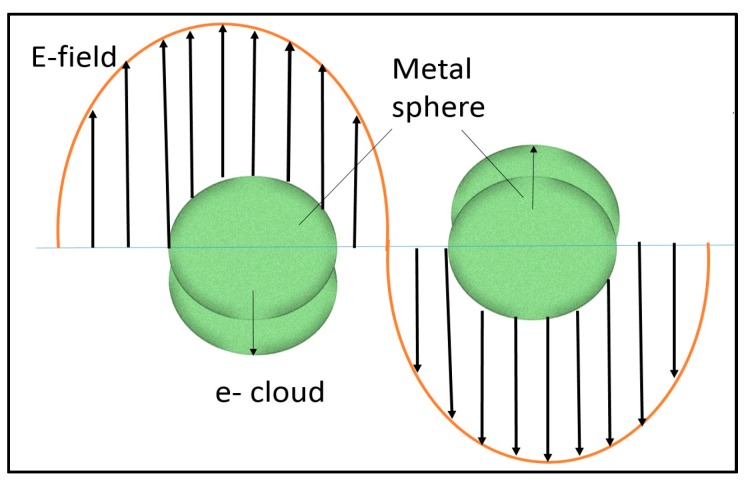
The schematic diagram is representing surface plasmon resonance in a spherical metal particle induced by the electric field component of incident light (adapted from [138]).

Dispersal of noble metal nanoparticles of size 10 to 100 nm into semiconductor photocatalyst shows significant enhancement in photocatalytic activity under UV and visible range irradiation. In a conducting material, plasmons are the collective oscillation of the free charge (Figure 4). The oscillations confined to the surfaces of conducting materials are called surface plasmons, which strongly interact with light. When the real part of the dielectric function goes to zero at the plasmon frequency, a resonance in the absorption occurs.

The strong interaction with the resonant photons through an excitation of SPR is the characteristic of plasmonic metal nanoparticles. As such, SPR can be defined as the collective oscillation of valence electrons induced by the resonant photon. Au, Ag and Cu nanoparticles show resonant behavior when irradiated by UV and visible photons. As a large fraction of the solar energy consist of UV-vis photons, these noble materials become more promising [103]. This resonance frequency can be turned by manipulating the size, shape, material, and proximity of the nanoparticles [135,136,137].

For example, the plasmon resonance of silver lies in the UV range but can be shifted to the visible range by minimizing the size of its nanoparticles; in the case of the gold, a smaller size of the nanoparticle can shift the plasmon resonance from the visible range to the infrared range [31]. Plasmonic metal nanoparticles exhibit the exceptional capability of concentrating electromagnetic fields, scattering electromagnetic radiation, and converting the energy of photons into heat, which is useful for different applications [103].

### 4.1. Fundamental of Plasmonic Photocatalyst

The photocatalytic reaction itself is a very complex process; the addition of the plasmonic resonance of noble metal nanoparticles makes it more complicated. The understanding of the physical mechanism of plasmonic photocatalysis is progressing steadily but has not reached unanimity. It is generally accepted that the vital role is played by the energy transferred from the metal nanoparticles to the semiconductors. However, the difference lies in the detailed approach of energy transfer in exciting more number of electrons and holes [30].

The presence of noble metal nanoparticles benefits photocatalysis in different ways. The two very distinct characteristics of plasmonic photocatalysts are SPR or LSPR and a Schottky junction. Even though Schottky junction is not a plasmonic or resonance effect but it is considered as an intrinsic feature while discussing plasmonic photocatalysts. When noble metal nanoparticles come in contact with the semiconductor photocatalysts, it results in the Schottky junction, which builds an internal electric field in a region (space–charge region) inside the photocatalyst, closer to the metal–semiconductor interface. Once the electrons and holes are generated near the Schottky junction, this internal electric field would force them to move in a different direction [128]. Moreover, a fast lane for charge transfer is provided by the metal part [139]; its surface also acts as a charge trap center and can host more active sites for the light-induced reaction. Both the Schottky junction and the fast-lane charge transfer help to minimize the electron–hole recombination process [30].

The surface plasmon resonance (SPR) of the noble metal nanoparticles in response to the incident light is the major attribute of the plasmonic photocatalyst. It brings enhancement in the photocatalytic activity. Depending on the size, the shape, and the surrounding environment, the resonance frequency of noble metal nanoparticles (like Au/Ag) can be tuned to fall in the visible or near UV range [138]. When it falls in the visible light range, the large bandgap photocatalyst such as TiO_2_ becomes visible-light responsive. Again, for low-bandgap photocatalysts like Fe_2_O_3_ [140], SPR can significantly enhance the visible light absorption and UV absorption for large bandgap photocatalysts [127]. This feature is very useful for weakly absorbing materials. Due to the strong absorption, the larger portion of the incident light is absorbed by the photocatalyst surface in a thin layer (~10 nm) providing a shorter distance between the photo-generated electrons and/or holes and the surface, thus making it comparable to shorter carrier diffusion length [73,103,140]. This effect helps materials with poor electron transport. It also contributes in exciting more number of electrons and holes [70,71,72], increasing the rate of redox reaction and the mass transfer by heating up the surrounding environment [141,142,143] and enhancing adsorption by polarizing non-polar materials [142].

In general, the photocatalysis process consists of five individual steps [144,145], starts with reactants transfer to the photo-reactant surface, adsorption of the reactants, followed by redox reaction in this adsorbed phase, then product desorption from the surface, and finally, transfer of product away from the surface. Plasmonic photocatalysts contribute to all these steps. The enhancement in the creation and separation of excited electrons and holes increases the redox reaction rate, the SPR, the Schottky junction, the metal’s fast transfer, charge carrier trapping, and large contact surface has a significant influence here [127,139,140,146,147,148,149]. The heating effect also increases the redox reaction rate [141,142,143,150,151], and benefits the reactant transfer, product desorption and enhancing fluid mixing by product boosting. The polarization enhances the adsorption of reactants [142]. These are the major impacts of plasmonic photocatalysts that had been identified and verified so far [30], which explain how plasmonic photocatalysts mostly show great enhancement in the photocatalytic activity.

### 4.2. Reduction of CO_2_ with H_2_O by Plasmonic Photocatalyst

Noble metal nanoparticles in plasmonic photocatalysts generally coupled with substrates having a larger surface area and active sites. Thus in a co-operative way both the noble metal nanoparticle and the substrate work to enhance the photocatalytic activity [32]. The size, shape, and distribution of noble metal nanoparticles do have an effect on the plasmonic oscillation [152,153,154]. In this section, some of the recently studied plasmonic photocatalysts (Au/Ag), that demonstrate a significant enhancement in the photocatalytic CO_2_ reduction with H_2_O are being discussed.

#### 4.2.1. Au Deposited TiO_2_

In a comprehensive study of photocatalytic CO_2_ reduction with water, Hou et al. found that depositing Au nanoparticles on top of TiO_2_ results in plasmonic enhancement [155]. In the visible range (532 nm) of light, the photon energy matches the plasmon resonance of the Au nanoparticles; at this wavelength, a 24-fold enhanced photocatalytic activity was reported.

**Figure 5:**
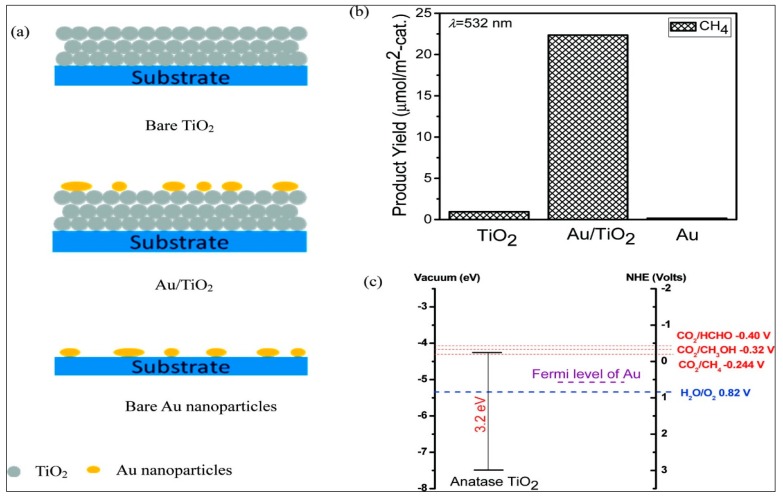
(**a**) Schematic diagrams of bare Au, Au/TiO_2_ and the bare TiO_2_ photocatalysts, (**b**) amount of CH_4_ formed on these photocatalyst surfaces after 15 h and (**c**) the relevant redox potentials of CO_2_ and H_2_O under visible light and energy band positions of anatase TiO_2_ and Au (Adapted from [155]).

The strong electric fields created by SPR of the Au nanoparticles locally excite the electron–hole pairs in the TiO_2_ at a rate several times higher than that by usual incident light; this phenomenon is considered to be responsible for this plasmonic enhancement. The mechanisms of photocatalytic CO_2_ reduction by Au nanoparticle/TiO_2_ were investigated under two visible and two UV range wavelengths to separate the contribution of plasmon resonance from the effect of electronic transitions in Au on the overall process. Three basic types of sample were used in this study: (1) bare TiO_2_; (2) Au nanoparticles deposited on top of TiO_2_; and (3) bare Au nanoparticles. A quantitative study of the reaction products was conducted to determine the mechanism behind the higher photocatalytic activity. Hou et al. suggested an interband transition hypothesis for the contribution of Au nanoparticles in TiO_2_ for the increase in photoactivity. This hypothesis was based on the comparative energies of the electrons and holes of the solid material with the redox potentials of the reaction product. Figure 5b shows that for all the three types of sample, CH_4_ is the only detected product. The product formation in photocatalytic CO_2_ reduction with H_2_O under visible light (532 nm) irradiation on Au/TiO_2_ is significantly high.

The explanation for this reaction is obtained by comparing the reduction potentials of the possible products from CO_2_ reduction with the energies of VB and CB of TiO_2_ [92,156], as shown in Figure 5c. The reduction potential of CO_2_/CH_4_ lies under the CB of TiO_2_ [157], but for other possible products, i.e., HCOH and CH_3_OH, it lies above the CB potential of TiO_2_ [92,156]. This fact indicates the CH_4_ formation is energetically favorable for photocatalytic reduction of CO_2_ by TiO_2_. The reaction scheme for this photocatalytic process is as follows, where $e_{cond}^{-}$ symbolizes an electron in the CB and $p_{val}^{+}$ symbolizes a hole in the valance band (VB) [92]:

$$\mathrm{Photocatalyst}+h\upsilon \to \mathrm{Photocatalyst}*(e_{cond}^{-}+p_{val}^{+})$$

Oxidation reaction

$$H_{2}O+2p_{val}^{+}\to \frac{1}{2}O_{2}+2H^{+}$$

Reduction reaction

$$CO_{2}\;(\mathrm{aq})+2H^{+}+2e_{cond}^{-}\to \mathrm{HCOOH}$$

$$\mathrm{HCOOH}+2H^{+}+2e_{cond}^{-}\to \mathrm{HCHO}+H_{2}O$$

$$\mathrm{HCHO}+2H^{+}+2e_{cond}^{-}\to CH_{3}\;\mathrm{OH}$$

$$CH_{3}\;\mathrm{OH}+2H^{+}+2e_{cond}^{-}\to {\mathrm{CH}}_{4}+H_{2}O$$

To initiate the reduction process for CH_4_ formation, electrons from the CB of TiO_2_ are transferred to CO_2_ [158]. In the case of bare TiO_2_-photocatalyzed CO_2_ reduction, the product yield is very low because the energy of 532 nm wavelength light (2.41 eV) is considerably lower than the band-gap of TiO_2_ (3.2 eV). For the third type of sample (i.e., bare Au), the amount of product formation is almost negligible, thus suggesting the importance of photocatalytic TiO_2_ surface in this reduction process. These results correlate with their previous publications [159,160]. Under visible-light irradiation, the sub-bandgap transitions in TiO_2_ generate electron–hole pairs not that in Au. When the photon energy is sufficiently high to excite d-band electrons of Au to its CB, which lies above the CB of TiO_2_ in the UV range (254 nm), a different mechanism takes place, resulting in the formation of additional products, including C_2_H_6_, CH_3_OH, and HCHO. As the energy of d-band excited electrons lies above the redox potentials of CO_2_/C_2_H_6_, CO_2_/CH_3_OH, and CO_2_/HCHO, these additional products are formed [155].

#### 4.2.2. Ag Supported on AgIO_3_

**Figure 6:**
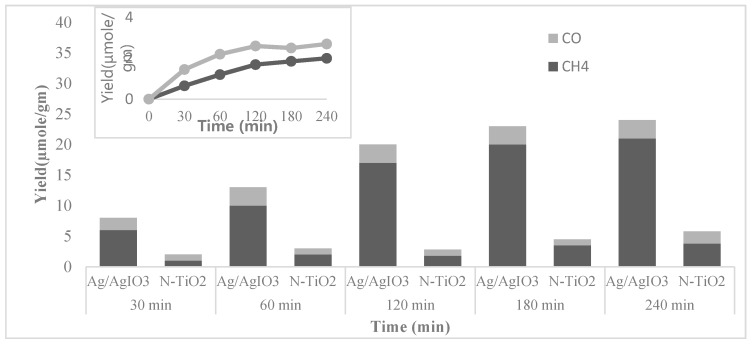
Schematic diagram showing time dependence yields of CH_4_ and CO yields under visible light irradiation over Ag/AgIO_3_ particles and over N doped-TiO_2_. The inset shows the time dependence of CH_4_ and CO yields over AgIO_3_ under UV-vis light (Reproduced from [57]).

In the photocatalytic conversion of CO_2_ using H_2_O, the plasmonic photocatalyst Ag supported on AgIO_3_ (Ag/AgIO_3_ particles) displays high activity and stability. In a longitudinal study, He et al. reported the synthesis, characteristics, and application of this plasmonic photocatalyst in CO_2_ reduction with H_2_O, where CH_4_ and CO are produced under visible-light irradiation (>400 nm wavelength) [57]. It was found that Ag plasma induced photo-excited electrons in AgIO_3_ facilitate the reduction reaction. The comparative photocatalytic activities towards the CO_2_ reduction of a different photocatalyst in the presence of water under visible light were evaluated by the amount of carbon containing products. Figure 6 shows the increasing amount of CH_4_ and CO formation with time under visible light range.

In comparison to N-TiO_2_, Ag/AgIO_3_ particles display higher photocatalytic reaction rate for CH_4_ and CO production. The result of this 240 min reaction indicates the significance of Ag nanoparticles on AgIO_3_ in photocatalytic CO_2_ reduction. Under visible light irradiation, the estimated quantum yield is 0.19% for CO_2_ reduction on Ag/AgIO_3_ catalysts. The turnover number (TON) are 1367 and 167, respectively, for CH_4_ and CO formation at 240 min. This information leads to the assumption that each Ag atom exposed to visible light is a potential active site. In photocatalytic CO_2_ reduction, photoexcitation of Ag electrons to higher energy state is the initial step as AgIO_3_ itself cannot be excited by visible light. The free electrons in Ag are either promoted by the intraband transitions from the half-filled s band below the Fermi level via surface plasmon excitation to unfilled s band states above the Fermi level or by the interband transition from the d-band to unfilled s-band states [57]. The first interband excitation occurs in Ag nearly at 3.8 eV energy band [161]. However, the energy of light in the visible range (400 nm wavelength) is 3.1 eV, which is less than the required amount of energy for interband transition in Ag; thus, electrons cannot be excited to the ECB (the CB edge potential) from the d band. Thus, the interband transition is not possible. This signifies that the SPR of Ag nanoparticles is the cause of photocatalytic reduction of CO_2_ in this process. The plasmonic electrons and holes cannot drive the oxidation and reduction half-reactions because the plasmonic charges exist in the Fermi energy of the metal [31]. Therefore, in this process, both oxidation and reduction half-reactions occur on the AgIO_3_ surface.

The contribution of SPR in CO_2_ reduction by using Ag/AgIO_3_ under visible light irradiation was established by studying the wavelength dependence quantum yield. When the light-excited plasmon produced energy or charge was transferred to AgIO_3_ to drive the photocatalysis, only at that time photocatalytic activity at the SPR wavelength was reported [162]. In AgIO_3_ the electron–hole pairs are generated by dipole-dipole interaction during resonant energy transfer from Ag to AgIO_3_ and direct electron transfer between Ag (donor) and AgIO_3_ (acceptor) [162,163]. These photo-excited electrons lead to the formation of CO by photocatalytic reduction of CO_2_, where the photo-excited holes lead to the formation of O_2_ by reacting with H_2_O. He et al. also ran 10 repeated reaction cycle under visible light irradiation with Ag/AgIO_3_ plasmonic photocatalyst to examine its stability, where the catalyst showed almost constant photocatalytic activity each time [45]. Their study demonstrated that “Ag/AgIO_3_ particles manifest high and stable photocatalytic activity in the conversion of CO_2_ to CH_4_ and CO using water vapor.”

#### 4.2.3. Ag Supported on Ag_2_SO_3_

The plasmonic-semiconductor structure of Ag supported on Ag_2_SO_3_ was also mentioned as an effective photocatalyst for photocatalytic reduction of CO_2_ with water vapor under visible light irradiation by the seminal work of Wang et al. [62]. The major carbon-containing product from this CO_2_ reduction is CH_4_, with a small amount of CO. The quantum yield is 0.126%, with an energy returned on energy invested of 0.156%. The study suggested that the energy conversion from incident photons to SPR oscillations of Ag nanoparticles initiates the photocatalytic activity of the catalysts. Ag_2_SO_3_ obtained this plasmonic energy by either one or both direct electron transfer and resonant energy transfer. The energy transfer results in separation of photogenerated electron–hole pairs, thereby increasing electron density and transferring the SPR electrons from Ag to the CB of Ag_2_SO_3_ by a direct electron transfer process as it lifts the Fermi level of Ag. Moreover, by dipole–dipole interaction, resonant energy transfer from Ag could also generate electron–hole pairs in the Ag_2_SO_3_. Thus, for the photocatalytic CO_2_ reduction, the light-induced sites are provided by Ag nanoparticles and the reaction sites are provided by Ag_2_SO_3_.

**Figure 7:**
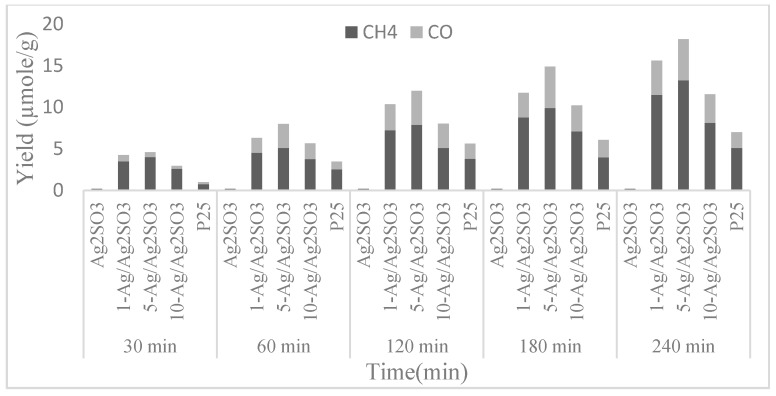
Formation of the product over TiO_2_ (P25), Ag_2_SO_3_, and Ag/Ag_2_SO_3_ photocatalysts under visible light irradiation as a function of time (Reproduced from [62]).

Figure 7 reveals the yield of products (CH_4_ and CO) in the presence of Ag_2_SO_3_, 1-Ag/Ag_2_SO_3_, 5-Ag/Ag_2_SO_3_, and 10-Ag/Ag_2_SO_3_ as a function of time. The CO yield on 1-Ag/Ag_2_SO_3_, 5-Ag/Ag_2_SO_3_, and 10-Ag/Ag_2_SO_3_ reached 3.06, 4.94, and 2.44 μmol/g, respectively, and the amount of CH_4_ formation reached 10.55, 12.05, and 7.42 μmol/g, respectively. Therefore, the optimal catalyst is 5-Ag/Ag_2_SO_3_. This result can be explained in terms of surface coverage of Ag_2_SO_3_ by Ag nanoparticles, where more Ag nanoparticles provide more light-induced sites, whereas fewer reaction sites result in a decrease in the rate of CO_2_ reduction. For practical applications, evaluating the stability of photocatalyst is an important concern. In this case, Ag/Ag_2_SO_3_ is considered as a stable photocatalyst for CO_2_ reduction because it performed consistently under visible light irradiation even after 10 repeated catalytic cycles. The XRD patterns and surface atomic compositions of new and used catalyst are also quite indistinguishable [62], thus confirming its stability

## 5. Conclusions

From this review, it can be concluded that the environmental challenges are no longer confined issues but become global problems involving climate changes. To solve these problems, we are still far from having a scientific as well as a cost-effective photocatalyst for photocatalytic CO_2_ reduction with H_2_O. As previously mentioned, both physical mechanism and product distribution in photocatalytic reactions are very complicated which confines its applicability. The present situation in this area of research is quite confusing, and comparing the efficiency of the different photocatalysts is also difficult due to the high variability of influencing factors and reaction conditions. Details such as mass balance (moles of CO_2_ converted into the specific product), product distribution, and the amount of reducing agent, time requirement, solution pH, temperature, CO_2_ pressure, light power, and activity decay over time were not discussed in many of the studies. Comprehensive studies on this process for further movement towards its practical implementation are necessary. An all-in-one standard format that will be unified and widely accepted should be established.

The low photocatalytic efficiency, low response to sunlight, inefficient electron transport between reduction and oxidation catalysts, and high recombination rate of photogenerated species are the major difficulties responsible for the current considerably low rate of average productivity in photocatalytic reduction of CO_2_ with H_2_O. Another drawback is the short lifetimes of one-electron-reduced species and the photo-excited state in the presence of O_2_ generated by H_2_O oxidation. Despite the fact that UV light can supply more energy than visible light, the availability of visible light from the abundant natural sunlight makes visible-light-harvesting photocatalysts the most desired ones for this process. In its infancy, plasmonic photocatalysts already showed promising performance to overcome the first two shortcomings of the above list. The boundaries of these highly efficient noble-metal photocatalysts are expanding rapidly that it is realistic to expect that plasmonic photocatalysts will contribute significantly in future environmental remedies. Further research should focus on the fabrication of optimal structured visible-light-responsive photocatalysts with a wide bandgap, high rate of photogenerated electron–hole transport, and low rate of recombination, to increase the possibility for practical application of photocatalytic CO_2_ reduction with H_2_O.

## Figures and Tables

**Table 1:** Advances in photocatalytic systems for CO_2_ reduction with water since the year 2010.

Photocatalyst	Radiation Source	Major Products	Comments	References
0.5 wt % Cu/TiO_2_-SiO_2_	Xe lamp (2.4 mW cm^−2^, 250–400 nm)	CO and CH_4_	The synergistic combination of Cu deposition and high surface area of SiO_2_ support enhanced CO_2_ photoreduction rates.	[36]
ZnGa_2_O_4_	300 W Xe arc lamp	CH_4_	Strong gas adsorption and large specific surface area of the mesoporous ZnGa_2_O_4_ photocatalyst contribute to its high photocatalytic activity for converting CO_2_ into CH_4_.	[37]
(RuO + Pt)-Zn_2_GeO_4_	300 W Xe arc lamp	CH_4_	In the presence of water, ultra-long and ultrathin geometry of the Zn_2_GeO_4_ nano-ribbon promotes CO_2_ photo-reduction, which was significantly enhanced by loading of Pt or RuO_2_.	[38]
Ag/ALa_4_Ti_4_O_15_ (A = Ca, Ba and Sr)	400 W Hg lamp	CO, HCOOH, and H_2_	On the optimized Ag/BaLa_4_Ti_4_O_15_ photocatalyst, CO was the reported as the main product. The molar ratio of O_2_ production (H_2_ + CO:O_2_ = 2:1) demonstrated that water was consumed as a reducing reagent in the photocatalytic process.	[39]
I-TiO_2_ nanoparticles	450 W Xe lamp	CO	High photocatalytic activity was observed under visible light and the efficiency of CO_2_ photoreaction was much greater than undoped TiO_2_ due to the extension in the absorption spectra of TiO_2_ to the visible light region and facilitated charge separation.	[40]
LiNbO_3_	Natural sunlight or Hg lamp (64.2 mW cm^−2^)	HCOOH	The MgO-doped LiNbO_3_ showed an energy conversion efficiency rate of 0.72% which was lower than that for the gas–solid catalytic reaction of LiNbO_3_ (2.2%).	[41]
G-Ti_0.91_O_2_ hollow spheres	300 W Xe arc lamp	CH_4_, CO	The presence of G nanosheets compactly stacking with Ti_0.91_O_2_ nanosheets allows the rapid migration of photo-generated electrons from Ti_0.91_O_2_ nanosheets into G and improves the efficiency of the photocatalytic process.	[42]
Graphene oxides (GOs)	300 W commercial halogen lamp	CH_3_OH	Among all GOs, GO-3 exhibited the highest efficiency as a photocatalyst for CO_2_ reduction under visible light, and the conversion rate of CO_2_ to CH_3_OH on modified GO (GO-3) was 0.172 mmol g^−1^ cat h^−1^, which is six-fold higher than that of pure TiO_2_.	[43]
W_18_O_49_	300 W Xe lamp	CH_4_	The oxygen-vacancy-rich ultrathin W_18_O_49_ nanowires can be used to design materials with extraordinary photochemical activity because it displayed high CO_2_ reduction capability in presence of water.	[44]
Zn_1.7_GeN_1.8_O	300 W Xe arc lamp	CH_4_	Zn_1.7_GeN_1.8_O loaded with co-catalysts showed significantly higher conversion rate of CO_2_ into CH_4_.	[45]
Pt-, Au-, or Ag-loaded mesoporous TiO_2_	350 W Xe lamp	CH_4_	The mesoporous TiO_2_ showed higher efficiency towards CO_2_ reduction when loaded with noble metal particles, and the order of enhanced photocatalytic activity was Pt > Au > Ag. The optimum loading amount of Pt was 0.2 wt %.	[16]
0.5 wt % Pt loaded ZnAl_2_O_4_-modified mesoporous ZnGaNO	300 W Xe lamp (λ = 420 nm)	CH_4_	The high photocatalytic activity of this photocatalyst was attributed to the improved gas adsorption of the mesoporous structure, the chemisorption of CO_2_ on the photocatalyst and the narrow bandgap of ZnAl_2_O_4_-modified ZnGaNO to extend the light absorption.	[46]
Ga_2_O_3_ with mesopores and macropores	300 W Xe lamp (500 mW cm^−2^)	CH_4_	Ga_2_O_3_ with mesopores and macropores showed high photocatalytic activity due to its higher CO_2_ adsorption capacity (300%) and increased surface area (200%) compared to the bulk nanoparticles.	[47]
Pt-TiO_2_ thin nanostructured films	400 W Xe lamp	CO and CH_4_	The catalyst can be produced at an industrial scale for commercial application and showed high efficiency for selective CH_4_ formation.	[48]
HNb_3_O_8_	350 W Xe lamp	CH_4_	KNb_3_O_8_ and HNb_3_O_8_ were synthesized by the conventional solid-state reaction and performed more effectively in photocatalytic CO_2_ reduction than commercial TiO_2_.	[49]
ZnO-based materials	8 W fluorescent tube (average intensity 7 mW cm^−2^)	CO, CH_4_, CH_3_OH, H_2_	N-doping did not show any important influence on the photocatalytic behavior of ZnO-based photocatalysts. The mesoporous structure of ZnO favored CO and H_2_ production, but catalysts with Cu showed an enhancement in the hydrocarbon production, mainly CH_3_OH.	[50]
Ag, Pt, bimetallic Ag–Pt and core–shell Ag@silica (SiO_2_) nanoparticles with TiO_2_	100 W Hg lamp (330 nm)	CH_4_	The use of a reactor with three optical windows, a combination of both bimetallic co-catalysts, and Ag@SiO_2_ nanoparticles increased the product formation significantly compared to bare TiO_2_.	[51]
Carbon nanotubes Ni/TiO_2_ Nano-composites	75 W visible daylight lamp (λ > 400 nm)	CH_4_	Compared to Ni/TiO_2_ and pure anatase TiO_2,_ Ni/TiO_2_ incorporated with carbon nanotubes demonstrated maximum CH_4_ product yield of 0.145 mmol h^−1^ g^−1^ catalysts after 4.5 h of irradiation under visible light.	[52]
Pt/Cu/TiO_2_	200 W Xe lamp	CH_4_, CO, H_2_	The addition of co-catalyst Pt decreases the selectivity for CO_2_ photo-reduction; however, loading Cu onto TiO_2_ increases the selectivity from 60 to 80%.	[53]
Au/Pt/TiO_2_	500 W Xe lamp	CH_4_, CO	Plasmonic photocatalyst Au/Pt/TiO_2_ provided a more effective way to harvest solar energy by consuming a high-energy photon in the solar spectrum (UV region) and using it for charge carrier generation. Moreover, it also utilized visible light to enhance the photocatalytic activity.	[54]
20 wt % montmorillonite modified TiO_2_	500 W Hg lamp (365 nm)	CH_4_	Loading of montmorillonite on TiO_2_ enhanced the surface area and reduced particle size, thus improving charge separation, resulting in maximum yield for CH_4_ (441.5 mmol·g·cat^−1^ h^−1^).	[55]
0.5 wt % Pt/NaNbO_3_	300 W Xe lamp (λ > 300 nm)	CH_4_, CO, H_2_	The cubic-orthorhombic surface-junctions of mixed-phase NaNbO_3_ enhanced the charge separation, thereby improving its photoactivity.	[56]
Ag supported on AgIO_3_ (Ag/AgIO_3_ particles)	500 W Xe arc lamp	CH_4_ and CO	In the conversion of CO_2_ to CH_4_ and CO using water vapor, Ag/AgIO_3_ particles showed high and stable activity because of the surface plasmon resonance effect of Ag particles.	[57]
g-C_3_N_4_/NaNbO_3_ nanowires	300 W Xe arc lamp	CH_4_	An intimate interface formation was suggested between the C_3_N_4_ and NaNbO_3_ nanowires in g-C_3_N_4_/NaNbO_3_ heterojunction photocatalyst, resulting in almost eight-fold higher CO_2_ reduction than individual C_3_N_4_ under visible light irradiation.	[58]
In_2_O_3_/g-C_3_N_4_	500 W Xe lamp	CH_4_	The addition of In_2_O_3_ nanocrystals onto g-C_3_N_4_ surface improved the photocatalytic CO_2_ reduction process significantly due to the interfacial transfer of photo-generated electrons and holes between g-C_3_N_4_ and In_2_O_3._	[59]
SnO_2−x_/g-C_3_N_4_ composite	500 W Xe lamp	CO, CH_3_OH, and CH_4_	Enhancement in the surface area of g-C_3_N_4_ was observed by introducing SnO_2−x_. Improve photocatalytic performance was attributed to the increased light absorption and accelerated the separation of electron–hole pairs.	[60]
AgX/g-C_3_N_4_ (X = Cl and Br) nanocomposites	15 W energy-saving daylight bulb.	CH_4_	Under ambient condition and low-power energy-saving lamps, the optimal 30 AgBr/pCN (protonated graphitic carbon nitride photocatalyst) sample showed highest photocatalytic activity with significant enhancement in CH_4_ formation compared to individual AgBr and pCN photocatalyst.	[61]
Ag supported on Ag_2_SO_3_ (Ag/Ag_2_SO_3_)	500 W Xe lamp	CH_4_ and CO	Plasmonic photocatalyst Ag/Ag_2_SO_3_ was stable towards CO_2_ photoreduction after 10 repetitive catalytic cycles with high efficiency under visible light irradiation.	[62]

**Table 2:** Reduction potentials for the CO_2_ reduction process. E^0^: Standard reduction potential.

Reactions	E^0^/eV
CO_2_ + e^−^ → CO_2_	*≥−*1.9
CO_2_ + 2e^−^ + 2H^+^ → HCOOH	−0.61
CO_2_ + 2e^−^+ 2H^+^ → CO + H_2_O	−0.53
CO_2_ + 4e^−^ + 4H^+^ → HCHO + H_2_O	−0.48
CO_2_ + 6e^−^ + 6H^+^ → CH_3_OH + H_2_O	−0.38
CO_2_ + 8e^−^ + 8H^+^→ CH_4_ + 2H_2_O	−0.24

**Table 3:** Influence of reaction phase on photocatalytic reduction ^a^ of CO_2_ with H_2_O using TiO_2_ and 0.5 wt % Pt-TiO_2_ photocatalyst [107].

Reaction Mode	Photocatalyst	Formation Rate (μmol·g^−1^h^−1^)			R (Electron) (μmol·g^−1^h^−1^)	Selectivity for CO_2_ Reduction (%)
		CO	CH_4_	H_2_		
Solid–gas	TiO_2_	1.2	0.38	2.1	10	56
solid–liquid	TiO_2_	0.80	0.11	5.3	13	19
solid–gas	Pt-TiO_2_	1.1	5.2	33	110	40
solid–liquid	Pt-TiO_2_	0.76	1.4	55	123	11

^a^ Reaction conditions: catalyst, 0.020 g; CO_2_ pressure, 0.2 MPa; H_2_O, 4.0 mL; irradiation time, 10 h.

**Table 4:** CO_2_ reduction over ALa_4_Ti_4_O_15_ (A = Ca, Sr and Ba) photocatalysts with various co-catalysts ^a^ [39].

Photo-Catalyst	Band Gap/eV	Co-Catalyst (wt %)	Loading Method	Activity/μmol·h^−1^			
				H_2_	O_2_	CO	HCOOH
BaLa_4_Ti_4_O_15_	3.9	none	-	5.3	2.4	0	0
BaLa_4_Ti_4_O_15_	3.9	NiOx ^b^ (0.5)	impregnation	58	29	0.02	0
BaLa_4_Ti_4_O_15_	3.9	Ru (0.5)	photodeposition	84	41	0	0
BaLa_4_Ti_4_O_15_	3.9	Cu (0.5)	photodeposition	96	45	0.6	0
BaLa_4_Ti_4_O_15_	3.9	Au (0.5)	photodeposition	110	51	0	0
BaLa_4_Ti_4_O_15_	3.9	Ag (1.0)	photodeposition	10 ^c^	7.0 ^c^	4.3 ^c^	0.3 ^c^
CaLa_4_Ti_4_O_15_	3.9	none	-	1.3	0.6	0.07	0
CaLa_4_Ti_4_O_15_	3.9	Ag (1.0)	photodeposition	5.6	2.1	2.3	1.3
SrLa_4_Ti_4_O_15_	3.8	none	-	0.8	0.5	0.06	0
SrLa_4_Ti_4_O_15_	3.8	Ag (1.0)	photodeposition	2.7	1.8	1.8	0.5

^a^ Catalyst 0.3 g, water 360 mL, CO_2_ flow system (15 mL·min^−1^), a 400 W high-pressure mercury lamp, an inner irradiation quartz cell. ^b^ Pretreatment: Reduced at 673 K and subsequently oxidized at 473 K after impregnation (543 K for 1 h). ^c^ Initial activity.

**Table 5:** Effect of loading method of Ag co-catalyst on the photocatalytic activity for CO_2_ reduction over ALa_4_Ti_4_O_15_ (A = Ca, Sr, and Ba) ^a^ [39].

Photocatalyst	Loading Amount/wt %	Loading Method	Activity/μmol·h^−1^			
			H_2_	O_2_	CO	HCOOH
BaLa_4_Ti_4_O_15_	1.0	Impregnation ^b^	8.2	5.7	5.2	0.2
BaLa_4_Ti_4_O_15_	1.0	Impregnation ^b^ + H_2_ red ^c^	5.6	8.7	8.9	0.3
BaLa_4_Ti_4_O_15_	0.5	Liquid-phase reduction	4.5	6.8	11	0.03
BaLa_4_Ti_4_O_15_	1.0	Liquid-phase reduction	5.6	12	19	0.4
BaLa_4_Ti_4_O_15_	2.0	Liquid-phase reduction	10	16	22	0.7
BaLa_4_Ti_4_O_15_	3.0	Liquid-phase reduction	9.7	14	19	0.1
BaLa_4_Ti_4_O_15_	5.0	Liquid-phase reduction	4.8	6.6	12	0.02
BaLa_4_Ti_4_O_15_	1.0	Liquid-phase reduction	20 ^d^	11 ^d^	0 ^d^	0 ^d^
SrLa_4_Ti_4_O_15_	1.0	Liquid-phase reduction	4.8	5.8	7.1	0.8
CaLa_4_Ti_4_O_15_	1.0	Liquid-phase reduction	3.2	6.6	9.3	0.4

^a^ Catalyst 0.3 g, water 360 mL, CO_2_ flow system (15 mL·min^−1^), a 400 W high-pressure mercury lamp, an inner irradiation quartz cell. ^b^ 723 K for 1 h, ^c^ 473 K for 2 h, ^d^ Ar flow.
